# Arginine-Based Inhibitors of Nitric Oxide Synthase: Therapeutic Potential and Challenges

**DOI:** 10.1155/2012/318087

**Published:** 2012-09-04

**Authors:** Jan Víteček, Antonín Lojek, Giuseppe Valacchi, Lukáš Kubala

**Affiliations:** ^1^International Clinical Research Center-Center of Biomolecular and Cell Engineering, St. Anne's University Hospital Brno, 656 91 Brno, Czech Republic; ^2^Institute of Biophysics, Academy of Sciences of the Czech Republic, 612 65 Brno, Czech Republic; ^3^Department of Evolutionary Biology, University of Ferrara, 44100 Ferrara, Italy; ^4^Department of Food and Nutrition, Kyung Hee University, Seoul 130-701, Republic of Korea

## Abstract

In the past three decades, nitric oxide has been well established as an important bioactive molecule implicated in regulation of cardiovascular, nervous, and immune systems. Therefore, it is not surprising that much effort has been made to find specific inhibitors of nitric oxide synthases (NOS), the enzymes responsible for production of nitric oxide. Among the many NOS inhibitors developed to date, inhibitors based on derivatives and analogues of arginine are of special interest, as this category includes a relatively high number of compounds with good potential for experimental as well as clinical application. Though this group of inhibitors covers early nonspecific compounds, modern drug design strategies such as biochemical screening and computer-aided drug design have provided NOS-isoform-specific inhibitors. With an emphasis on major advances in this field, a comprehensive list of inhibitors based on their structural characteristics is discussed in this paper. We provide a summary of their biochemical properties as well as their observed effects both *in vitro* and *in vivo*. Furthermore, we focus in particular on their pharmacology and use in recent clinical studies. The potential of newly designed specific NOS inhibitors developed by means of modern drug development strategies is highlighted.

## 1. Introduction

Nitric oxide (NO) is a crucial signaling molecule in vertebrates. NO is generated by NOSes. In vertebrates, three isoforms of NOS have been identified: endothelial NOS (eNOS, also referred as NOS3), neuronal NOS (nNOS, also referred as NOS1), and inducible NOS (iNOS, also referred as NOS2). The endothelial isoform is expressed constitutively in the endothelium lining of blood vessels. Neuronal NOS is constitutively expressed as well and primarily located in the central nervous system and skeletal and heart muscle cells. Inducible NOS is expressed in macrophages and some other cell types upon their activation by a wide range of proinflammatory stimuli. The activity of eNOS and nNOS is controlled by the calcium level via calmodulin-calcium interaction. Conversely iNOS has calmodulin bound permanently; this is why its function depends solely on the expression level [1, 2].

**Figure 1:**
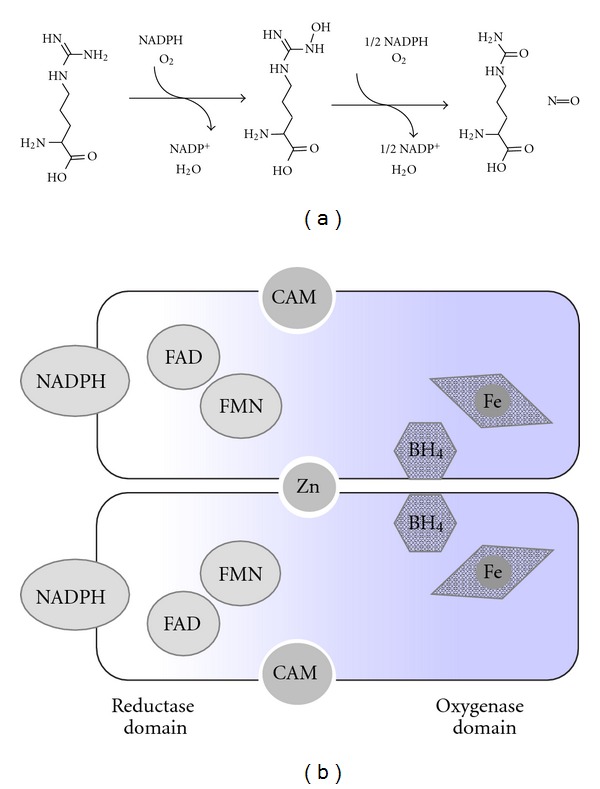
(a) Scheme of the two-step oxygenation of arginine catalysed by NO synthase. (b) Scheme of the mature NOS dimer: Each subunit contains tetrahydrobiopterin (BH_4_) and heme near the active site of the oxygenase domain (grey). The reductase domain (white) houses the NADPH-binding site as well as two-electron transfer cofactors (flavine adenine dinucleotide FAD and flavine mononucleotide FMN). The zinc bound at the interface mediates dimerisation. Calmodulin (CAM) binding regulates the activity.

All NOSes metabolize L-arginine to L-citrulline and NO via two consecutive NADPH-dependent monooxygenations (Figure 1(a)). The active NOS consists of two identical monomers, each one having reductase and oxygenase domains. The reductase domain contains a NADPH-/NADP- binding site and a two-component (FAD, FMN) electron transport chain to deliver reducing equivalents to the oxygenase domain, which is linked with a polypeptide segment incorporating a calmodulin-binding site. The oxygenase domain contains heme at the bottom of an arginine-binding pocket, which forms the active site. Further, tetrahydrobiopterin cofactor is located at the interface of subunits near the heme. The linkage of subunits is stabilized due to a zinc ion and tetrahydrobiopterin bound at the interface (Figure 1(b)) [1, 2]. All three isoforms contain highly conserved catalytical cores. Indeed, 16 out of 18 amino acid residues within a 6 Å distance from the active site of the oxygenase domain are conserved among mammalian NOSes with known structure [3].

NO was primarily described to act in the cardiovascular system as a key regulator of vascular tone. Beyond this function it can prevent platelet activation, limit leukocyte adhesion to the endothelium, and regulate myocardial contractility [4]. Further, NO can act as a neurotransmitter in glutamatergic nerves in the central and peripheral nervous systems [5]. Finally, NO is requisite in immune system reactions. It is involved in microbicidal mechanisms of macrophages. On the other hand, despite NO playing beneficial roles, it can also be involved in numerous pathological situations such as hypotension accompanying septic shock, essential hypertension, and atherosclerosis [4]. The increased formation of NO was found to have neurotoxic effects and can contribute to the pathogenesis of stroke and other neurodegenerative disorders [5]. In general, the overwhelming production of NO contributes to the pathogenesis of both acute and chronic inflammatory processes, and NO has been recognized as one of the main signaling molecules involved in these processes [6].

Due to the importance of NO in various pathological processes (see above), NOSes are regarded as an important pharmacological target and a great deal of effort has been made to design specific NOS inhibitors [7]. However, the high degree of similarity among NOS isoforms poses an obstacle when attempting to find a specific inhibitor of a particular isoform. Up to this time, a wide range of NOS inhibitors have been described. From a historical perspective the first compounds considered as NOS inhibitors were simple arginine derivatives and close analogues. Then a trial and error approach was applied when undertaking the substitution of scaffolds with a potential to inhibit NOS. Once a broad set of compounds with the capacity to inhibit NOS was known and computer modeling was available, the pharmacophore approach was utilized extensively. This computational approach is based on the determination of the minimal structure of an inhibitor (i.e., the pharmacophore) to achieve optimal binding to NOS. Then compounds having such pharmacophore are screened for their ability to inhibit NOS. When the structural data of NOS were made available, many inhibitors were designed by means of computer-aided virtual screening of large libraries of compounds [8–10]. Since the active sites of NOSes are highly conserved, the virtual screening approach has been improved by taking into account the conformational plasticity of the target protein [11]. The pharmacophore approach was improved as well to get so-called fragment hopping, which capitalizes on scaling down the pharmacophore to a cluster of atoms, virtual graphs, and/or vectors. Thus delicate differences among NOS isozymes may be utilized to generate inhibitors [12].

Inhibitors of NOSes can be classified according to various points of view. The classical enzymological approach allows us to distinguish reversible inhibitors (subgroups: competitive, uncompetitive, noncompetitive and mixed type), irreversible inhibitors, as well as reaction-based inhibitors, whose action is dependent on the enzymatic reaction. The most widely used classification of inhibitors relies on the identification of the site of inhibitor binding to the NOS enzyme (described in detail elsewhere), allowing four different classes of inhibitors to be recognized. The first class, virtually the largest class of NOS inhibitors, interacts with the arginine-binding site. Some compounds belonging to this class are reaction-based inhibitors, as they require an active enzyme and NADPH to proceed to full inhibition. The second class includes a set of compounds that mimic tetrahydrobiopterin cofactor. The third class consists of inhibitors interacting directly with the heme. Several inhibitors belonging to this class bind to the heme of a monomer form of the enzyme and prevent formation of the enzyme active dimer. The fourth class covers NOS inhibitors interacting with calmodulin or flavine cofactors [1]. A structural point of view may also be taken into consideration, dividing NOS inhibitors into two principal groups: amino acid-based and nonamino-acid-based inhibitors. The first group covers derivatives and close analogues of arginine. The second group includes a broad range of compounds not having the arginine-like amino acid scaffold.

The goal of this paper is to provide a comprehensive list of inhibitors based on arginine, together with a summary of their observed effects both *in vitro* and *in vivo* with a focus on their employment in clinical studies.

## 2. The Literature Review

### 2.1. Simple Arginine Derivatives

Primarily, simple arginine derivatives were first considered as inhibitors for experimental use because they were expected to compete with arginine for the active site of NOS. Indeed such expectation has been fulfilled in general. Moreover, some members of this group of inhibitors can act as reaction-based inhibitors as well.

#### 2.1.1. L-N^*ω*^-Methylarginine (L-NMA)

L-NMA, also referred to as N^G^-monomethyl-L-arginine (L-NMMA), occurs naturally in living organisms, as it is a product of the degradation of arginine-methylated proteins [13]. L-NMA is one of the first compounds which were intuitively employed to inhibit NOSes at the end of the eighties [14–16]. L-NMA has been used widely as a general tool to decrease NO bioavailability or to establish the NO dependency of a physiological process. This has led to the discovery of the physiological function of NO by means of *in vitro* models (reviewed extensively e.g., [17–19]). Since the structure of L-NMA is very close to arginine, it acts as a competitive inhibitor of all NOSes. Further, it can behave as a reaction-based inhibitor of iNOS and nNOS but not eNOS (Table 1) [20]. Inducible NOS and nNOS slowly metabolize L-NMA in a NADPH- and BH_4_-dependent manner [20] to form N-hydroxyderivative that is either processed to L-citrulline and NO or inactivates NOS because of heme loss. The partition ratio of L-citrulline and NO formation and inactivation is about 100 : 1 [21].

Considering the Ki values, L-NMA concentrations to inhibit NOSes effectively ranged from 10 to 100 *μ*mol/L for an experiment with extracts or purified enzymes (Table 1). When treating cells and tissues, the effective concentration ranged from 0.1 to 10 mmol/L. L-NMA is of low toxicity and very stable. It is not degraded by arginase in the urea cycle [22]. These are the main advantages for experimental work, together with the fact that L-NMA could be taken up easily through the action of cationic amino acid transporters, as L-NMA has about the same affinity to arginine transport systems as L-arginine [23].

**Pharmacokinetics**

Pharmacokinetics studies in rats and dogs treated with L-NMA (1.7 mg/kg i.v. and 25 to 100 mg/kg i.v. bolus) revealed rapid distribution and nonlinear L-NMA elimination from plasma but minimal loss in feces and urine [24, 25]. A distribution study showed the highest accumulation of L-NMA in the spleen of rats, and the distribution in the brain pointed to a preference for the olfactory bulb and cerebellum. In the bovine brain there was a higher accumulation in cerebellar gray matter, the hippocampus, and the hypothalamus [26]. Pharmacokinetic assay in septic patients infused with L-NMA hydrochloride (1 to 20 mg/(kg h) for up to 8 hours) revealed that renal clearing plays a major role at low infusion rates. In contrast, at infusion rates equal to or higher than 5 mg/(kg h), removal due to tissue uptake plays the major role, indicating progressive NOS inhibition as well as nonlinear pharmacokinetics at such L-NMA dosages [27].

**Animal Studies**

A wide range of animal studies were performed employing L-NMA to determine the physiological role of NO in immune (e.g., [17]), nervous (e.g., [19]), and cardiovascular (e.g., [18, 28]) systems and particularly as a potential treatment of septic shock to reverse sepsis-associated hypotension. Primarily, Kilbourn et al. found that L-NMA (20 mg/kg, i.v.) prevented hypotension in septic dogs in an arginine-reversible manner, though L-NMA increased blood pressure in control animals [29, 30]. In further studies, L-NMA (up to 300 mg/kg) always reduced sepsis-associated hypotension and increased systemic vascular resistance, yielding frequently improved survival rates (reviewed by [31]). However, side effects including decreased cardiac output, decreased oxygen delivery due to attenuated local blood, and even increased mortality were observed in some cases. These controversies were suggested to be attributed to different dynamics of sepsis in different animal models, together with different levels of dosage and different timings of the intervention with L-NMA [31].

**Human Studies**

Despite certain controversies in animal models, many clinical trials have been carried out using L-NMA. Nearly all of them were performed employing L-NMA hydrochloride (also referred to as “546C88”). When administered to healthy volunteers, L-NMA (3 mg/kg, i.v. and 0.03 to 1.0 mg/kg/min for 3 min, i.v.) decreased heart rate, stroke volume, and consequently cardiac output, increased vascular resistance and blood pressure as well as pulmonary vascular resistance, but did not increase pulmonary artery pressure [32, 33]. L-NMA has been used as a tool to modulate hemodynamics in a healthy volunteer-based study aimed at muscle oxygen uptake at the onset of moderate intensity exercise [34].

Since animal studies have revealed the possibility to attenuate the symptoms of septic shock with L-NMA, the use of this compound to treat this pathological condition in clinical practice has attracted a great deal of attention. In a very early study, L-NMA (0.3 to 1.0 mg/kg, i.v.) showed a dose-dependent increase in systemic vascular resistance and blood pressure in a patient with septic shock [35]. In the next placebo-controlled study by these authors, L-NMA (0.3 to 1.0 mg/kg, i.v.) induced a widespread increase in vascular tone and raised blood pressure. However, such intervention produced a fall in cardiac output [36]. Further, L-NMA (1 to 20 mg/(kg h) for 8 h, i.v.) reduced or even eliminated the necessity of using norepinephrine (a vasopressor) to maintain blood pressure above 70 mmHg without adverse effects in a study with 32 septic shock patients [37]. Next, in a phase II clinical trial with 312 severe septic shock patients, L-NMA (5 to 20 mg/(kg h), i.v. for up to 72 hours) confirmed the possibility of withdrawing the vasopressor and maintaining blood pressure above 70 mmHg, together with the possibility of promoting sepsis resolution but with a survival rate similar to the placebo group [38]. In similar settings, L-NMA treatment reduced plasma levels of nitrate, indicating a lowered bioavailability of NO, and allowed the substitution of conventional vasoconstrictor agents [39]. In a phase III trial aimed at efficacy and safety over a longer period of time, 797 patients with septic shock received either placebo or L-NMA (2.5 to 20 mg/(kg h) for 7 or 14 days, i.v.). Such treatment resulted in a lower incidence of deaths caused by multiple organ failure. However, the overall mortality compared to placebo was 10% higher due to a higher proportion of cardiovascular deaths in the L-NMA-treated group, indicating adverse effects of this compound on the cardiovascular system over a longer period. Thus, the study was terminated [40].

L-NMA was also tested for its therapeutic potential in the treatment of migraine and chronic tension-type headache, based on the hypothesis that NO may be involved in pain induction. The research group of J. Olesen showed that L-NMA (6 mg/kg, i.v.) brought relief from migraine and chronic tension-type headache symptoms in two placebo-controlled trials [41, 42]. However, a search for the mechanism of migraine attack and the possibility of its modulation by L-NMA revealed that the basal tone of human cerebral arterioles but not of conduit arteries is NOdependent, suggesting a more complex antimigraine action of L-NMA than a simple vasoconstriction [43–45].

Recently thirteen clinical trials employing L-NMA have been designed (see supplementary Table 1 in Supplementary Materials available online at doi:10.1155/2012/318087). These studies were focused on cardiovascular function, and L-NMA was used as a tool to modulate NO synthesis. The results of these studies are, however, not publicly available.

#### 2.1.2. L-N^*ω*^,N^*ω*^-Dimethylarginine (ADMA)

Another naturally occurring inhibitor is L-N^*ω*^,N^*ω*^-dimethylarginine (commonly referred to as asymmetrically dimethylated arginine, ADMA). Similarly to L-NMA, ADMA is a product of the degradation of arginine-methylated proteins [13]. Though its presence in animals was discovered much earlier, its biological importance was demonstrated at the beginning of the nineties [46, 47].

ADMA is considered as a nonspecific competitive inhibitor of NOSes. Though the inhibition of NO synthesis with ADMA has been well studied in cells (IC_50_ value about 10 *μ*mol/L, reviewed [48]) the data with purified enzymes are sparse and contradictory. Komori [49] found ADMA to be a weak inhibitor of iNOS and nNOS (*K*_*i*_ > 300 *μ*mol/L in both cases). On the other hand a later work suggested ADMA to be a potent inhibitor of nNOS with a submicromolar inhibition constant (*K*_*i*_) [50] (Table 1). Its experimental application as a tool to modulate NOS activity is very limited. Nearly all ADMA-related studies performed with cells, tissues, and animals are focused on elucidation of the pathological role of ADMA in cardiovascular diseases (reviewed in [51–55]). To our knowledge no data has been published which considers ADMA as a potentially clinically applicable NOS inhibitor.

#### 2.1.3. L-N^*ω*^-Nitroarginine (L-NNA) and L-N^*ω*^-Nitroarginine Methyl Ester (L-NAME)

L-NNA (also referred to as N^G^-nitro-L-arginine) is one of the first synthetic NOS inhibitors. Its ability to block NO synthesis was recognized in the early nineties [56, 57]. Initially L-NNA was recognized as a competitive inhibitor of all NOSes having high selectivity to eNOS and nNOS over iNOS [58]. A later study, however, indicated only minor selectivity to nNOS and eNOS [59]. Such a pattern of selectivity correlates with a different mechanism of L-NNA binding to individual isoforms. Though L-NNA interacts with all NOSes noncovalently, its coupling with iNOS is immediate and rapidly reversible with arginine. However, the binding of L-NNA to eNOS and nNOS is a time-dependent process with a relatively slow reversal [60, 61]. L-NNA shows excellent stability in aqueous environments and low toxicity. The only limiting factor of L-NNA application in biological systems is poor solubility (about 4 mmol/L) at neutral pH. This disadvantage was addressed by introducing another compound L-N^**ω**^-nitroarginine methyl ester (L-NAME). This compound may act as a weak NOS inhibitor, but it is readily hydrolyzed by ubiquitously present esterases to L-NNA in a biological system [22]. The application of L-NAME in experiments *in vitro* and *in vivo* provides an advantage over L-NNA, as there is no major limitation on solubility in an aqueous environment. Taking the Ki values in the low micromolar range into consideration (Table 1), the effective concentration of L-NNA in an experiment with tissue extracts and purified enzymes goes up to 100 *μ*mol/L. The use of L-NNA with cells, tissues, and experimental animals may benefit from the specific method of inhibitor distribution. Since the nitration of the guanidinium moiety in L-NNA restricts the overall positive charge, this inhibitor is transported as a neutral amino acid. Thus, the variable extracellular concentration of cationic amino acids including L-arginine should not influence the accumulation of L-NNA significantly [62]. Technical suggestions related to the use of L-NNA and L-NAME in cells and tissues are summarized in Griffith and kilbourn1996 [22].

Both L-NNA and L-NAME have been extensively used in experimental practice. Thus only selected examples are presented herein [63–68]. Among numerous studies employing L-NNA and L-NAME in *in vitro* experiments, L-NNA and L-NAME were used to determine the role of NO in leukocyte adhesion [63]. L-NNA was also used to determine the dependency of neuronal cell death on NO in primary brain cultures [64] and cerebellar granule neurons cocultured with lipopolysaccharide-stimulated microglial cells [65]. In experiments with endothelial cells, L-NAME inhibited angiogenesis under chemical [67] or growth factor [66] stimulation, demonstrating the significance of NO in this process. L-NNA together with L-NMA was used to demonstrate the role of NO in cardiac muscle cell physiology [68].

**Pharmacokinetics**

The pharmacokinetic properties of L-NNA were extensively studied in rats [69]. It showed a biphasic pharmacokinetic profile with a terminal half life of 20 hours after an intravenous bolus of 20 mg/kg. L-NNA showed distribution volume 2.2 L/kg. A steady state concentration up to 30 *μ*g/mL in plasma was shown to be achievable using an initial bolus and maintenance infusion of L-NNA. Under these conditions, organs like the liver and kidney as well as muscle accumulated L-NNA to the highest extent. On the other hand, the concentration of L-NNA in cerebrospinal fluid was found to be five times lower than in plasma although the brain tissue itself contained a similar level of L-NNA as that found in plasma [69]. The distribution of L-NNA in brain tissue studied by means of ^3^H L-NNA found a predominant presence of L-NNA in the rat accessory olfactory bulb, the amygdaloid complex, the Islands of Calleja, and the cerebellum [70]. In rat cerebellum the binding of ^3^H L-NNA was arginine reversible, having nanomolar-affinity-binding sites [71]. Solid evidence that L-NAME has to be hydrolysed to L-NNA to have full inhibitory effect *in vivo* was found, having the implication that the increase in coronary perfusion pressure in isolated rat hearts was achieved faster with L-NNA compared to L-NAME. However, the final level was the same in both cases [72].

In humans the attention of pharmacokinetics studies was focused on L-NAME. This compound was shown to be hydrolysed to L-NNA in whole blood with a half life of 30 minutes in an *ex vivo* experiment [72]. Such a process was even faster *in vivo*. Mainly L-NNA (~6 *μ*mol/L) remained in plasma after 90 minutes following L-NAME intravenous infusion into healthy humans at 4 mg/kg over 60 minutes. This indicated the half life of L-NAME to be about 12 min. Such treatment resulted in L-NNA muscle content of 38 *μ*mol/kg (dry weight) and caused about 70% inhibition of NOS activity in muscles 30 minutes after the L-NAME infusion [73]. Similarly, a study with patients suffering from sepsis infused with L-NAME at 1 mg/(kg h) for 12 hours revealed extensive hydrolysis of this compound with a half life of 19 minutes. The maximal concentration of L-NNA in the plasma of septic patients reached about 6 *μ*g/mL and decayed slowly showing a half life of 23 hours. Distribution volumes were 0.45 and 1.96 L/kg for L-NAME and L-NNA, respectively, indicating that L-NNA penetrates the tissues effectively after being formed in plasma [74].

**Animal Studies**

L-NAME and to a lesser extent L-NNA have both been broadly used in animals and only a brief selection of these studies can be mentioned here [31, 98–100]. Primarily, L-NAME has been established as a tool to modulate hemodynamics. The intravenous application of L-NAME (20 mg/kg) caused complex changes in blood flow in dogs [98]. Local bolus administration of L-NAME (1 to 30 mg) into the uterine artery in ewes caused the reversal of the estradiol-induced increase in uterine blood flow [99]. The application of L-NNA and L-NAME up to 0.1 mmol/L to the guinea pig coronary bed attenuated or reversed the effect of a set of vasodilators (bradykinine, 5-hydroxytriptamine, etc.) [100]. Similarly to L-NMA, L-NAME was mostly tested to treat septic shock symptoms in various animal models (reviewed by Kilbourn et al. [31]). In general, treatment with L-NAME (up to 100 mg/kg) resulted in the reversal of sepsis-associated hypotension and an increase in systemic vascular resistance, and, in some case, an increase in survival rate. However, in comparison to L-NMA, a larger number of studies pointed to adverse effects of L-NAME application. These negative effects included decreased cardiac output, lowered regional blood flow, and finally, an increase in mortality in several cases. However, a certain degree of controversy has been attributed to differences among various septic models together with differences concerning the dosage and timing of the intervention with L-NAME.

**Human Studies**

In human clinical trials, both L-NNA and L-NAME were used to modulate hemodynamics in septic shock pathological conditions. In a very early study, L-NAME showed a dose-dependent increase in systemic vascular resistance and blood pressure in a patient with septic shock [35]. Next, L-NNA (bolus 20 mg/kg, i.v.) increased systemic vascular resistance and blood pressure in 8 septic patients but decreased cardiac index. These changes were reversed with arginine infusions (200 mg/kg) [101]. Similar results were obtained in a trial involving the prolonged infusion of L-NNA (1 mg/(kg h) for 12 hr, i.v.) into patients with septic shock. However, this work indicates the limited beneficial effects of such treatment, given that 7 out of a total of 11 patients died [102]. Aside from the treatment of sepsis, L-NAME was tested to treat asthma. L-NAME (2 mL of 100 mmol/L solution) administered by nebulization to healthy volunteers and patients with asthma did not show any adverse effects and the exhaled NO level dropped by about 50% in both groups [103], indicating the possibility of using this compound in asthma treatment.

Further, a set of six clinical trials was designed recently employing L-NNA or L-NAME in order to modulate cardiac physiology and hemodynamics (see Supplementary Table 2). Their results are, however, not publicly available.

#### 2.1.4. Higher L-N^*ω*^-Alkylated Arginines

These compounds, which do not occur naturally, were synthesized in the nineties. They differ from L-NMA in elongated N^*ω*^-alkyl chain moiety, which may be unsaturated or cyclic. L-N^*ω*^-propylarginine (commonly referred to as L-NPLA), N^*ω*^-allyl-L-arginine, N^*ω*^-cyclopropyl-L-arginine, and N^*ω*^-propargyl-L-arginine belong to these types of inhibitors that are experimentally used. They allow for the formation of different types of radicals upon oxidation with NOS and have been mostly employed to elucidate the mechanisms of the reaction-based inhibition of iNOS and nNOS by L-NMA. These inhibitors all have low micromolar affinities to NOS. L-NPLA inhibits NOS reversibly with a strong preference for nNOS [75, 79] and may act as a reaction-based inactivator of nNOS as well. Further, N^*ω*^-cyclopropyl-L-arginine functions strictly as a nonselective competitive inhibitor of NOSes, whereas its stoichiometric isomer N^*ω*^-allyl-L-arginine brings about the reaction-based inactivation of iNOS and nNOS in addition to the nonselective competitive inhibition of NOSes [76, 77]. Interestingly, N^*ω*^-propargyl-L-arginine acts only as a potent nonselective competitive inhibitor of NOSes [75, 104]. Taken together, a small degree of manipulation with the N^*ω*^-alkyl substituent may largely affect the behavior of the arginine derivative in regard to selectivity and the mechanism of NOS inhibition.

Among these inhibitors, only L-NPLA has attracted some attention from life scientists. This compound is of low toxicity and due to its preference for nNOS it has been used as a tool to inhibit this isoform *in vitro* as well as *in vivo*. Considering the Ki values, the concentration of this compound can be in the low micromolar range in an experiment with extract or purified enzyme having a preference for nNOS (Table 1).

*In vitro* L-NPLA (0.1 to 10 *μ*mol/L) has been applied to inhibit nNOS-mediated motorneuron cell death in mouse-derived cell culture [105]. Caron et al. have used L-NPLA (100 *μ*mol/L) as a tool to determine the role of NO in the ethanol-induced upregulation of muscarinic acetylcholine receptors in human neuroblastoma cells [106]. Further, L-NPLA (up to 300 *μ*mol/L) has been shown to inhibit the production of NO in pituitary GH3 cells having an IC50 of 19 *μ*mol/L [107].

**Pharmacokinetics**

As far as we are aware, no pharmacokinetics study with NPLA, either in animals or humans, has been published.

**Animal Studies**

Considering *in vivo* animal studies, L-NPLA (10 mg/kg) was utilized to elucidate the interaction of the calcium pump (PMCA4b) and nNOS in heart-related signaling in mice [108]. L-NPLA (2 mg/kg) was also used to determine the role of nNOS in the induction of nerve growth factor-induced neck muscle nociception in mice [109]. L-NPLA (20 mg/kg) was shown to block the effects of phencyclidine on prepulse inhibition and locomotor activity in mice [110].

**Human Studies**

In a human trial, a cutaneous application of 5 mmol/L of L-NPLA to human skin helped to determine the contribution of nNOS to local warming or heat stress-induced cutaneous vasodilatation [111]. Though L-NPLA shows a certain potential for use in clinical practice it has not been used in further clinical trials. To our knowledge, there are no patents associated with the clinical application of this compound either.

#### 2.1.5. L-N^*ω*^-Aminoarginine

L-N^*ω*^-aminoarginine (also referred to as N^G^-amino-L-arginine) belongs to the group of early synthetic inhibitors, which were recognized at the end of the eighties [112, 113]. It is a nonselective competitive inhibitor of NOSes showing low micromolar affinities (Table 1, [59]). However, it may act as a reaction-based inactivator as well (Table 1, [78]). Considering its affinity to NOSes (see Table 1), the effective concentration for an experiment with an extract or purified enzyme ranges up to tens of *μ*mol/L.

N^*ω*^-amino-L-arginine was used extensively to block NO release from endothelial cells and aortic rings (reviewed by [22]).

As far as we are aware, no complex pharmacokinetic study has been undertaken with N^*ω*^-amino-L-arginine, either in animals or humans. This results from the fact that N^*ω*^-amino-L-arginine acts as a convulsant. Further, N^*ω*^-amino-L-arginine shows an arginine-irreversible toxicity in awake animals. These facts rendered N^*ω*^-amino-L-arginine to be not an attractive NOS inhibitor for animal studies (reviewed by [22])

### 2.2. Arginine Peptides and Peptidomimetics

Since NOSes can process certain arginine containing dipeptides [114], efforts have been made to design synthetic arginine-based dipeptide inhibitors. Initially, the work by Silverman et al. in 1997 introduced dipeptides based on L-/D-nitroarginine coupled with L-/D-phenylalanine. These dipeptides were alkylated at the C-terminus to reduce polarity [80]. Among these compounds D-phenylalanyl-N^*ω*^-nitro-D-argininemethylester exhibited a strong effect on nNOS and eNOS but did not affect iNOS significantly (Table 1) [80]. In the further search for nNOS inhibitors, C-terminal alkylation has been substituted with the amide group, and other amino acids than D-phenylalanine have been coupled with L-NNA. This effort has resulted in a library containing 152 compounds. Among them, N^*ω*^-nitro-L-arginyl-L-diaminobutyramide has been shown to be a potent and selective inhibitor of nNOS (Table 1) [79, 115]. Conformationally restricted analogues of N^*ω*^-nitro-L-arginyl-L-diaminobutyramide having a cyclic proline-based moiety bound to the arginine amide group possess only a slightly increased potency to inhibit nNOS, as demonstrated by a representative compound N^*ω*^-nitro-L-arginyl-trans-3-amino-L-prolineamide (Table 1) [81, 116]. The potency of such N^*ω*^-nitro-L-arginine-based compounds to inhibit nNOS was retained or even slightly improved if the peptide bond was reduced, as in the case of (4S)-N-(4-amino-5-[aminoethyl]aminopentyl)-N′-nitroguanidine [82, 116]) or N-(4S)-{4-amino-5-[2-(2-aminoethyl)-phenylamino]-pentyl}-N′-nitroguanidine (Table 1) [83, 116]. Using computer-aided design approach called fragment hopping (see chapter: Current methods of inhibitor design among arginine-based NOS inhibitors) a set of 20 compounds mimicking the nitroarginine-based peptides was prepared. These inhibitors show submicromolar potency and selectivity to nNOS and more drug-like properties [12]. Most of the above-mentioned peptides and peptidomimetics are competitive inhibitors of NOSes. Their submicromolar potencies to nNOS and very good selectivity over other NOS isoforms give them a great potential for experimental and clinical applications. Since these compounds were mostly engineered to be of reduced polarity (see above) they are expected to penetrate living cells easily. The above-mentioned compound N-(4S)-{4-amino-5-[2-(2-aminoethyl)-phenylamino]-pentyl}-N′-nitroguanidine was shown to be able to protect cultured neurons from hypoxia-induced apoptosis [9]. Accordingly, this compound attenuated fetal neurodegeneration under hypoxia in rabbits in a dose-dependent manner [9].

In parallel to the team of Richard B. Silverman, Nobunori Kobayashi and his colleagues synthesized thiocitrulline-based dipeptides in the late nineties. Among the developed compounds, S-methyl-L-thiocitrullinyl-L-phenylalanine showed the strongest inhibition of iNOS and nNOS (Table 1) [117, 118]. Later, using human recombinant enzymes, it was demonstrated to have a very good selectivity to iNOS over eNOS and mild selectivity to iNOS over nNOS. No further application with purified enzymes or extracts has been published. Considering the application in cells, this compound showed a strong potency to inhibit cytokinine-induced NO production in human colorectal adenocarcinoma cells at concentrations in units of *μ*mol/L [84].

As far as we are aware, no pharmacokinetic studies or clinical trials have been undertaken with any arginine peptides or peptidomimetics mentioned above.

### 2.3. Thiocitrullines

The byproduct of NOS catalyzed NO synthesis L-citrulline cannot affect the activity of NOS [119]. It can, however, fit to the active site. This feature predetermines this compound to be an appropriate scaffold for inhibitor design. In 1994, a research group led by Griffith substituted the carboxamide group oxygen with sulphur to get L-thiocitrulline (commonly referred to as L-TC) and its derivatives [120]. This dramatically increased the affinity to NOSes. A strong preference of L-thiocitrulline for nNOS has been demonstrated using rat enzyme [121], but this has not been proven in the case of recombinant human enzyme though these differences may be attributable to different assay conditions (Table 1) [59]. Spectral measurement of NOS having the L-TC moiety bound indicated a direct interaction with heme iron [121]. Introducing additional substitution by means of S-methylation (S-methyl-L-thiocitrulline, commonly referred to as L-SMTC) or S-ethylation even increased the potency to inhibit NOSes, and a moderate selectivity to nNOS was observed among human NOSes (Table 1) [60]. More recent data obtained with recombinant human enzymes, however, indicated rather low selectivity to nNOS in the case of L-SMTC [59]. The S-alkylation disables the direct interaction with heme iron [122]. Further modifications introducing an alkyl substituent to the 3-position in the carbon chain of the amino acid yielded neither increased affinity nor selectivity to NOSes [123]. Regardless of the slightly different mode of interaction of L-thiocitrulline and S-alkyl-L-thiocitrullines with the NOS, they are all competitive inhibitors [60, 121].

L-TC as well as its methyl and ethyl derivatives have been successfully applied in experimental practice. They are readily soluble in an aqueous environment and stable except for alkalic solutions [22]. Considering the Ki values (see Table 1), the effective concentration to block activity in a purified enzyme or extract is in the range of 10 to 100 *μ*mol/L in the case of L-thiocitrulline and from 0.1 to 10 *μ*mol/L in the case of its methyl and ethyl derivatives. Experimental use of these compounds with cell and intact tissue covers comparable applications with a similar efficiency as L-NMA (reviewed by [22]). Interestingly, the arginine levels within cells and tissues are unlikely to affect their uptake given that L-thiocitrullines are neutral amino acids and are not likely to be transported by cationic amino acid transporters (see discussion in [121]).

**Pharmacokinetics**

A simple distribution study performed by Zhang et al. with ^11^C incorporated L-SMTC showed a rapid distribution over many organs in rats [124]. However, this compound was rapidly cleared from all parts except the liver, muscle, and the brain. In rat brains, L-SMTC distributed preferentially in the cerebellum and olfactory bulb. In baboons, uniform distribution in the gray and white matter of the brain was observed, but higher activity was detected in the region of the olfactory bulb. The surrounding tissue of the brain exhibited a higher amount of L-SMTC than the brain itself. As far as we are aware, no complex pharmacokinetic study of L-thiocitrullines has been carried out in animals or humans.

**Animal Studies**

Primarily, L-thiocitrullines were employed as nonspecific NOS inhibitors. L-thiocitrulline at a concentration of 20 mg/kg i.v. increased blood pressure in normotensive and septic rats [121]. Further, L-SMTC (20 mg/kg i.v.) caused the almost immediate reversal of blood pressure drops in septic rats and dogs and brought about an additional modest improvement in cardiac output as well [122]. L-SMTC was also used as a specific tool to determine biological functions of nNOS, though this may be somewhat speculative due to the incoherency of data on selectivity to nNOS (see above) [125–129]. Thus, L-SMTC (100 *μ*mol/L, intracerebroventricular application) was used to determine the role of nNOS in the modulation of sympathetic activity in vagotomized anesthetized pigs [125]. Further, L-SMTC (100 mg/kg i.p.) was employed to determine the contribution of nNOS in formation of cued and contextual long-term memory in mice [126]. In addition, the importance of nNOS for renal microvascular function [127] and hemodynamics as well as kidney pathogenesis under diabetes [128, 129] was suggested based on the application of L-SMTC (10 *μ*M; 0.1–0.5 mg/kg i.v. or 0.05 mg/kg into abdominal aorta).

To our knowledge no clinical trial conducted with thiocitrullines has yet been published or registered.

### 2.4. Amidino Aminoacids

Except for naturally occurring N^5^-(1-iminoethyl)-L-ornithine (L-NIO, also referred to as N^G^-iminoethylornithine), amidino amino acids are synthetic compounds that were recognized as NOS inhibitors in the nineties. These compounds contain an amidine group, an analogue of the guanidinium moiety of arginine. It is based on the substitution of one terminal amine of the guanidinium group with a hydrocarbon moiety.

The first analogue of arginine belonging to this group is N^5^-(1-iminoethyl)-L-ornithine (L-NIO), which is a potent nonselective competitive inhibitor of NOS (Table 1) [57, 130]. Further, L-NIO may cause reaction-based inactivation of nNOS as well [85]. A deeper characterization was undertaken with its derivative N^5^-(1-imino-3-butenyl)-L-ornithine (vinyl L-NIO, also referred to as L-VNIO), which may also act competitively and by means of a reaction-based mechanism as well. In both cases vinyl L-NIO shows a low micromolar affinity with a good degree of selectivity to nNOS (Table 1). The inactivation of nNOS by vinyl L-NIO is accompanied by loss of heme. The detailed mechanism is not clear yet but appears to be dependent on the unsaturated vinyl moiety, as the saturated derivative N^5^-(1-imino-3-butyl)-L-ornithine does not cause any inactivation despite similar affinities in the low micromolar range [85].

Further, a set of synthetic amidine aminoacids having side alkyl chain of the amidine moiety substituted with heteroatoms resulted from computer-aided design approach called virtual screening (see the chapter Current methods of inhibitor design among arginine-based NOS inhibitors). When these inhibitors were tested with purified NOSes one of them N^5^-[2-(methylthio)-1-iminoethyl]-L-ornithine showed the best potency and good selectivity to nNOS [86].

 The next interesting compound from this group is a synthetic homologue of L-NIO N^6^-(1-iminoethyl)-L-lysine (L-NIL), which was developed in the nineties. It is a moderately selective inhibitor of iNOS and has not been found to cause NOS inactivation (Table 1). This compound has similar chemical properties to L-NIO [131]. Later, a more stable and less hygroscopic prodrug of L-NIO, N^6^-(1-iminoethyl)-L-lysine 5-tetrazole-amide (L-NIL-TA, also referred to as SC-51), was introduced. This compound itself does not inhibit NOSes but is metabolized rapidly to release L-NIL (see below) [132]. L-NIO and its derivatives show a reasonable stability in an aqueous environment [22, 131]. Their effective concentration to inhibit NOS activity in purified enzyme or an extract ranges up to ~100 *μ*mol/L with the advantage of preferential inhibition of nNOS in the case of vinyl L-NIO and N^5^-[2-(methylthio)-1-iminoethyl]-L-ornithine (Table 1) and iNOS in the case of L-NIL. Except of N^5^-[2-(methylthio)-1-iminoethyl]-L-ornithine with no further application they have been broadly employed with cells. Here, we show a brief example of the application of these compounds. L-NIO was shown to be a potent inhibitor of NO synthesis, reaching maximal inhibition within 10 minutes of preincubation in experiments with rat and murine phagocytes [130]. Further, this compound served to determine the pivotal role of NO in *Leishmania major* killing in murine macrophages [133]. L-NIO also helped to determine the inhibitory role of NO in platelet adhesion under flow conditions [134]. In addition, it was used as a tool to limit NO synthesis by tumor cells [135].

L-NIL (up to 30 *μ*mol/L) was used to reveal the NO dependency of mesangial cell elimination by macrophages in murine [136]. L-NIL (up to 25 *μ*mol/L) also served to determine the iNOS dependency of the expression of glial fibrillary acidic protein (an important marker of neurodegeneration) in activated primary murine astrocytes [137]. As L-NIL-TA requires hydrolysis to release L-NIL and this hydrolysis was localized to blood [138], it is not surprising that this compound has not been applied in *in vitro* experiments with cells.

**Pharmacokinetics**

As far as we are aware there has been no study describing the pharmacokinetics of L-NIO or its derivatives. The pharmacokinetics of L-NIL have been included in studies of its prodrug L-NIL-TA. The oral and intravenous administration of ^14^C L-NIL-TA at 5 mg/kg to rats resulted in maximal plasma concentration 6.45–7.07 *μ*g/g equivalents within about 20 minutes, showing a decay with a half life of 63 and 81 hours, respectively [139]. The prodrug was rapidly hydrolysed to L-NIL, and the experimental evidence indicates that the primary site of hydrolysis is blood. Free L-NIL showed throughout the distribution in the body. Further, a complex metabolization of L-NIL occurred and the products was mostly the subject of renal clearing [138]. Concerning pharmacokinetics in humans, we are not aware of the published results of any pharmacokinetic study of any of the compounds belonging to the category of inhibitors.

**Animal Studies**

L-NIO (15–30 mg/kg, s.c.) attenuated colonic microvascular injury provoked by endotoxin when applied after the endotoxemia induction at a time when the expression of iNOS appears (i.e., a few hours after endotoxin administration). However, the early administration of L-NIO after endotoxin treatment had a rather harmful effect, most likely due to eNOS inhibition, suggesting the nonspecific action of L-NIO [140]. On the other hand, the specificity of L-NIL (L-NIL-TA) was suggested extensively. Thus, in a rat model of adjuvant induced arthritis, L-NIL administered orally (0.1 mg/mL in drinking water or a bolus of 100 mg by a gavage) reduced plasma levels of nitrite and joint inflammation without affecting blood pressure [141]. A study employing L-NIL (4 mmol/L, drinking water) in mice has demonstrated iNOS to be a critical factor in limiting tuberculosis spread [142]. Further, application of L-NIL reduced carrageenan-induced hindpaw edema in rats. Proving the specificity, L-NIL was only efficient at later time points after the carrageenan application when the expression of iNOS was elevated [143]. In a study employing a model of osteoarthritis in dogs, L-NIL (10 mg/kg orally, twice daily) reduced the clinical signs of osteoarthritis as well as the synovial inflammation [144]. Finally, oral application of L-NIL (4 mmol/L in drinking water) has improved memory and reduced amyloid pathology in mice [145]. Cancer-related studies using L-NIL-TA indicated that selective inhibition of iNOS may serve as chemoprevention of cancer (reviewed by [146]). Further, L-NIL-TA (10 mg/kg, p.o.) reduced exhaled NO and inhibited bronchial hyperresponsiveness in ovalbumin-challenged rats [147]. Treatment with NIL-TA (average 10 mg/(kg h), drinking water) for 14 days following ischemia almost completely prevented the loss of retinal ganglion cells in rats [148].

**Human Studies**

In humans, only L-NIL-TA was employed. It was tested to treat asthma. Oral administration of 200 mg of L-NIL-TA reduced exhaled NO levels in both healthy volunteers and asthmatics below 2 ppb, which represented more than a 90% reduction in asthmatics. The duration of the effect was at least 72 hours without affecting blood pressure, pulse rate, or respiratory function [149].

### 2.5. Aminoacid Sulphides, Sulphoxide, and Sulphones

Nonphysiological amino acids based on acetamidine derivatives of lysine and homolysine containing a sulphide, sulphoxide, or sulphone moiety were synthesized and introduced as NOS inhibitors in the nineties [87, 150]. Among them a sulphone derivative (2*R*)-2-amino-3-{[2-(ethanimidoylamino)ethyl]sulfonyl}propanoic acid (GW273629) and sulphide derivative (2*S*)-2-amino-4-{[2-(ethanimidoylamino)ethyl]thio}butanoic acid (GW274150) have been shown to be the most potent and selective to recombinant human iNOS (Table 1) [87, 88]. The binding of both GW273629 and GW274150 to NOS is competitive with arginine. The interaction with human eNOS and nNOS is immediate and reversible, but this is a time-dependent process in the case of human iNOS, which appears to be irreversible in the presence of NADPH [88].

Both GW273629 and GW274150 have good solubility and stability in water. Considering their affinities to NOSes, the effective concentration ranges from ~1 to ~50 *μ*mol/L with a strong preference for iNOS, though caution arises from the progressive NADPH-dependent inhibition of this isoform (Table 1) [87, 88].

Only a limited number of studies have employed these inhibitors *in vitro. *Primarily, the micromolar potency of GW273629 and GW274150 to iNOS and the high degree of selectivity to this NOS isoform have been verified in murine macrophages and in isolated rat tissue [88]. The distribution of these compounds within tissue is most likely mediated by y^+^L-type transporters as GW274150 has been found to be taken up by y^+^LAT-1 amino acid transporter in J774 murine macrophages [151]. GW273629 (10 *μ*mol/L) partially reversed LPS-induced hyporeactivity of rat superior mesenteric artery segments to phenylephrine [152]. GW274150 (10 *μ*mol/L) reduced shear stress-induced apoptosis in HUVEC cells, pointing to the role of iNOS in this process [153]. Further, GW274150 showed the effective inhibition of NO production in primary culture of rat proximal tubular cells in the range of 0.1 to 1000 *μ*mol/L [154] and was used to acquire evidence of the involvement of iNOS in the early differentiation of murine neurons [155].

**Pharmacokinetics**

GW273629 exhibited a biphasic pharmacokinetic profile with a terminal half life in plasma of about 3 hours in rats but only about 10 min in mice, where the pharmacokinetic profile was monophasic. By contrast, GW274150 had a biphasic pharmacokinetic profile with a terminal half life in plasma of about 6 hours in rats as well as mice. Such a difference in the half lives of these compounds was consistent with the remarkably higher distribution volume, and lower plasma clearance of GW274150, allowing for the longer-term effect of this compound. [88]. Both inhibitors are well tolerated when administered orally and their oral bioavailability reaches more than 90%. Consistently, the efficiency of GW274150 was almost the same for intraperitoneal and oral application in the case of application to LPS-treated mice (ED_50_ of 3.2 and 3.8 mg/kg, resp.) [88]. In addition, the fast uptake of orally administered GW274150 (30 mg/kg) was demonstrated as the plasma level peaked (128 *μ*mol/L) 60 minutes after application, reaching about half the maximal level within 10 minutes [156].

The study of the pharmacokinetics of GW274150 in humans has been included in at least two clinical trials (see Supplementary Table 3 trial numbers NCT00370435, NCT00379990); however, the pharmacokinetics data are not publicly available at present.

**Animal Studies**

Due to its better pharmacokinetic profile, GW274150 was preferred in a number of animal studies *in vivo*. In endotoxemic mice GW274150 (100 mg/kg, i.p.) caused significant inhibition of NO production, which lasted over 24 hours [88]. In hemorrhagic rats GW274150 (5 mg/kg, i.v.) attenuated renal dysfunction as well as liver and pancreatic injury but did not reduce a fall in blood pressure [157]. In a mouse model of arthritis induced by collagen, a daily administration of GW274150 (5 mg/kg, i.p.) reduced arthritis severity to the extent found in iNOS knockout mice [158]. GW274150 (5 mg/kg, i.v.) reduced renal dysfunction when applied 30 min prior to induction. In wild-type mice the renal dysfunction was attenuated to levels that have been observed in iNOS−/− mice under the same treatment, confirming the specificity of GW274150 treatment for iNOS inhibition [159]. In a rat model of inflammatory lung injury, GW274150 (2.5 to 10 mg/kg, i.p.) applied prior to the induction of inflammation by carrageenan suppressed the lung inflammatory reaction in a dose-dependent manner [160]. Similarly, in a mouse model of inflammatory bowel disease, GW274150 (5 mg/kg, i.p.) reduced the clinical signs of colitis [161]. In a rat model of inflamed paws, a single application of GW274150 (1 to 30 mg/kg, p.o.) dose ependently reduced nitrite accumulation in edema fluid and hypersensitivity to pain. Similarly, in a model of chronic injury to the sciatic nerve, GW274150 (3 to 30 mg/kg, p.o.) applied one hour prior to evaluation was effective in attenuating hypersensitivity to pain [156]. In a rat model of Parkinson disease induced with 6-hydroxydopamine, an administration of GW274150 (3 to 30 mg/kg, p.o.) twice daily showed significant neuroprotection. However, the efficiency of GW274150 exhibited a bell-shaped course as high doses of GW274150 were ineffective [162]. Taken together, GW274150 was demonstrated to be an excellent tool to reveal the involvement of iNOS in numerous pathophysiological processes. Regrettably, GW274150 is not commercially available due to patent restrictions at present [150].

**Human Studies**

In human clinical trials GW274150 showed a very good potential to attenuate inflammation-based pathological processes. Currently, the results of three clinical studies conducted at Glaxo Smith Kline have been published. In the first, patients with asthma diagnosed at least 6 month before the trial took (90 mg) once daily for 14 days. Such treatment reduced the exhaled NO level by about 70% but did not influence airway hyperreactivity or airway inflammatory cell numbers after an allergen challenge [163]. The other two clinical studies examined the potential of GW274150 to treat migraine. However, no reduction of migraine symptoms compared to placebo was observed, either when GW274150 was administered at doses ranging from 5 to 180 mg/kg as an early intervention in acute migraine [164] or when GW274150 was administered at doses of 60 or 120 mg/kg daily as a prophylactic agent for 12 weeks [165]. These two trials excluded the involvement of iNOS in migraine attack induction.

An additional two clinical trials with the goal of treating rheumatoid arthritis with GW274150 have been completed, but their results are not yet publicly available (see Supplementary Table 3).

### 2.6. Aromatic Amino Acids

In 2005, the research group of T. Higuchi introduced new synthetic inhibitors of NOS based on sulphide- or sulphone-containing amino acids modified with a pyridine-based moiety [89]. These compounds compete with arginine for the active site of NOS. Two of the most potent compounds which have arisen from this effort are of very strong potency as their *K*_*i*_ are at the level of units of *μ*mol/L. They, however, lack selectivity among individual NOS isoforms [89]. No experimental applications or patents involving these compounds have yet been published, and no clinical trials have been conducted.

### 2.7. Heterocyclic Amino Acids

These synthetic NOS inhibitors are based on a combination of ornithine scaffold, which can fit into the arginine-binding pocket [90], with imidazole, which is capable of binding the heme iron [166]. Among these compounds, *(S)*-2-amino-5-imidazolylpentanoic acid was shown to have good affinity to NOSes as the K_i_ values are at the level of a few units of *μ*mol/L but lacks selectivity to an individual NOS isoform (Table 1) [90]. Further, a set of heterocyclic analogues of thiocitrulline was synthesized. Among them, a compound having an ethylene bridge between sulphur and the terminal nitrogen of the isothiourea moiety (= aminothiazoline moiety) has been found to have a good affinity to NOS (*K*_*i*_ values at about 1 *μ*mol/L) but again shows no significant selectivity among NOSes (Table 1) [91]. Both above-mentioned heterocyclic amino acids have been proposed to interact with heme iron directly, but such interaction has not been verified experimentally [90, 91].

Due to lack of selectivity, these heterocyclic amino acid inhibitors have not attracted any further attention from pharmacologists. Thus, no experimental applications, patents, or clinical trials are associated with them.

### 2.8. Current Methods of Inhibitor Design among Arginine-Based NOS Inhibitors

**Figure 2:**
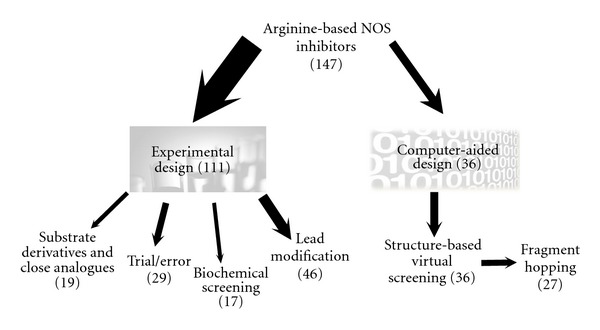
Summary of the use of current methods of inhibitor design among arginine-based NOS inhibitors. For a description of individual approaches see the chapter “Current methods of inhibitor design among arginine-based NOS inhibitors”. The width of arrows indicates the proportion in total number of arginine-based inhibitors. Only compounds for which quantitative data on the inhibition of at least one NOS isoform have been published were considered for this summary.

Up to date, a broad range of approaches to yield NOS inhibitors have been used. They include methods of experimental design as well as rapidly developing computer-aided design. Among 147 well-documented arginine-based inhibitors of NOS (i.e., inhibitors revealed in studies providing quantitative data on the inhibition of at least one NOS isoform), the major proportion (111) was revealed by experimental design methods, while the remaining number (36) was revealed by computer-aided design methods (Figure 2).

#### 2.8.1. Experimental Design

**Substrate Derivatives and Close Analogues**

Simple arginine derivatives and close analogues were intuitively taken as NOS inhibitors in early works. Because of their close similarity to arginine, they were anticipated to be competitive inhibitors. Such an approach yielded potent arginine-based inhibitors like L-NMMA, L-NAA, and L-NIO [14, 56, 112, 167]. However, this approach suffered from the coverage of a very narrow chemical space, which is not conceivable for modern drug design. In spite of this problem 19 simple arginine derivatives and close analogues have been well established as NOS inhibitors (Figure 2).

**Trial and Error Approach**

Further, a kind of trial and error approach allowed for the development of inhibitors with more variable scaffolds. Such an approach consists in the building of a hypothesis, the synthesizing of a potential inhibitor, and testing it. Though a wider chemical space may be covered, there is still the limitation of using compounds which have a certain degree of similarity to arginine or some already known NOS inhibitor. This is an early method of NOS inhibitor discovery but has not yet been abandoned as it has recently helped to reveal an inhibitor series with excellent potency and selectivity to iNOS [168]. The trial and error approach has so far yielded 29 well-documented arginine-based NOS inhibitors (Figure 2).

**Biochemical Screening**

In most cases the biochemical screening capitalizes on a large library of small organic compounds or their fragments (MW < 300 Da), which are tested in a high-throughput manner for the ability to bind the target or inhibit its enzymatic activity. A biochemical screening of a narrow group of compounds has been reported as well. Biochemical screening allows for the discovery of radically new inhibitor scaffolds with low similarity to substrates or already established inhibitors. The screening is carried out in a biochemical system harboring the target of a potential inhibitor. Among arginine-based NOS inhibitors, biochemical screening yielded a set of peptidomimetics [79] and GW274150, an inhibitor with the greatest potential for clinical application [87]. In total this approach has resulted in 17 well-documented arginine-based inhibitors of NOS (Figure 2).

**Cell-Based Screening**

In principle, cell-based screening works in the same manner as biochemical screening. The use of *in vitro* grown cells expressing the drug target brings the advantage of finding an inhibitor with more drug-like properties (e.g., low toxicity, good membrane permeability, etc.). This may in fact reduce the rate of failure in late stage pharmacological trials [169]. As far as we are aware, cell-based screening has not contributed to the discovery of any well-documented arginine-based inhibitor.

**Lead Modification**

Once a candidate inhibitor (a lead) is established it may be subjected to modification (addition or substitution of side chains or functional groups, etc.) in order to yield a compound with improved potency and selectivity to NOSes as well as better drug-like properties. Among well-documented arginine-based NOS inhibitors, lead modification contributed to the discovery of a set of peptides and peptidomimetics selective to nNOS [81–83, 170] and compounds having the 3-substituted arginine scaffold [123]. In total, this approach has yielded nearly one half (46) of all well-documented experimentally designed arginine-based NOS inhibitors.

#### 2.8.2. Computer-Aided Design

Once computers with reasonable capabilities were available they empowered inhibitor design greatly. In general, the computer-aided design of inhibitors allows for the screening (i.e., virtual screening) of a much larger number of compounds at much lower cost. Furthermore, the computer-aided quantitative structure activity relationship can reveal and exclude compounds having a potential for exhibiting unfavorable biological properties (toxicity, poor drug-like properties) [171].

**Virtual Screening**

Generally, there are two basic branches to virtual screening. These are ligand-based virtual screening and structure-based virtual screening. *Ligand-based virtual screening* relies on the assumption that similar molecules share similar properties. This implies that only knowledge of the structure of well-established ligands of a target molecule is sufficient to proceed. In practical terms, candidate inhibitors are found by searching for structurally similar compounds in a large compound library [171, 172]. *Structure-based virtual screening,* on the other hand, requires knowledge of the 3D structure of the pharmacophore. It proceeds by means of docking each individual member of a compound library with the target, with the criterion of having a maximal drop down of the free energy of the interaction between ligand and target. Ligands showing the highest decrease in the free energy (i.e., highest affinity) are chosen [171, 172].

Structure-based virtual screening of a small library was successfully applied to discover 9 (Figure 2) aminoacid amidine-based inhibitors of NOS [86]. The molecule to be docked can be scaled down to yield a simple fragment (molecular mass <300 Da). The fragments demonstrating close affinities to the target are then linked or hybridized together. Such an approach can largely reduce the computational time required to find a candidate inhibitor but presents the challenge of finding a suitable linker [173]. A modification of structural-based virtual screening of a fragment library called *fragment hopping* has also been developed. It consists in disassembling the pharmacophore to minimal pharmacophoric elements. For each such element, various fragments with different chemotypes can be found using a fragment and biostere library. This procedure allows for the exploration of a much wider chemical space and the utilization of delicate differences in the NOS active site that are responsible for isozyme selectivity. Indeed, application of this approach has resulted in 27 (Figure 2) nNOS selective inhibitors belonging to the peptidomimetics inhibitor class of arginine-based inhibitors [12, 174, 175].

**Computer-Aided Quantitative Structure Activity Relationship**

Despite being an integral part of the virtual screening effort, the *computer-aided quantitative structure activity relationship* [171, 176] may help to map the active/binding site of a target. Indeed, this has been shown in the case of NOSes, where this approach yielded intimate details of the active site [177] and tetrahydrobiopterin-binding site [178, 179].

## 3. Conclusions

In the past two decades, NOS inhibitors based on arginine have been of special interest, as this category includes a high number of compounds with a good potential for experimental as well as clinical application. Since these compounds are readily available and stable in an aqueous environment, they serve as a perfect tool to inhibit NOS experimentally.

Many of the first developed arginine-based inhibitors of NOS exhibit potent inhibition of NOS, low toxicity, and reasonable pharmacokinetics. The major problem consists in such inhibitors having no or low selectivity among NOS isoforms. In fact, this can result in pronounced side effects *in vivo*, hypertension and decreased cardiac output as a result of eNOS inhibition. Though the elevation of blood pressure due to an unspecific NOS inhibitor may be beneficial (e.g., in the treatment of septic shock), the overall adverse effects on the cardiovascular system are a big disadvantage. Despite these facts, the number of clinical trials using nonspecific arginine-based NOS inhibitors (L-NMA, L-NNA) keeps increasing since these compounds are an excellent tool for modulating hemodynamics.

The exceptional compound among the first developed arginine-based NOS inhibitors is L-NIL. It is of moderate selectivity to iNOS, well tolerated in animals and humans, and its prodrug L-NIL-TA allows for oral administration.

The application of modern inhibitor design approaches has resulted in selective NOS inhibitors. A broad range of peptides and peptide-mimicking compounds with high selectivity to nNOS have been designed by means of several approaches including a virtual screening method called fragment hopping. Some compounds of this set show good drug-like properties [9]. Further, biochemical screening among sulphide or sulphoxide-modified amidino aminoacid moieties has revealed an inhibitor called GW274150 with excellent selectivity to iNOS. This compound shows favorable pharmacokinetics and negligible toxicity and can be administered orally. Among all known inhibitors of NOS, GW274150 is the closest to approval for medical use.

Despite progress in the development of arginine-based NOS inhibitors, no clear clue for the NOS isoform selectivity of certain compounds (L-SMTC, L-NPLA, L-VNIO, L-NIL and GW274150) was obtained. These compounds interact, or are expected to interact, almost exclusively with the highly conserved substrate binding pocket of NOS, and no significant differences among NOS isoforms have been identified in this pocket [177]. In contrast, for selectivity of more voluminous NOS inhibitors, the importance of the pattern of hydrogen bonding with aminoacid residues or structural water at the entrance of the substrate binding pocket is suggested [9]. The conformational plasticity of the enzyme itself can play an important role as well [11].

Elucidation of the selectivity of the binding of small arginine-based NOS inhibitors remains a challenge. The increasing amount and quality of structural data on NOS, together with the rapidly improving precision of modern computer-aided structural analysis, suggest success in this field and offer a good chance of discovering new specific NOS inhibitors.

## Supplementary Material

The supplementary material provides a list of recently designed clinical trials using arginine-based nitric oxide synthase inhibitors with unpublished results.

## Figures and Tables

**Table 1:** List of the most important arginine-based inhibitors.

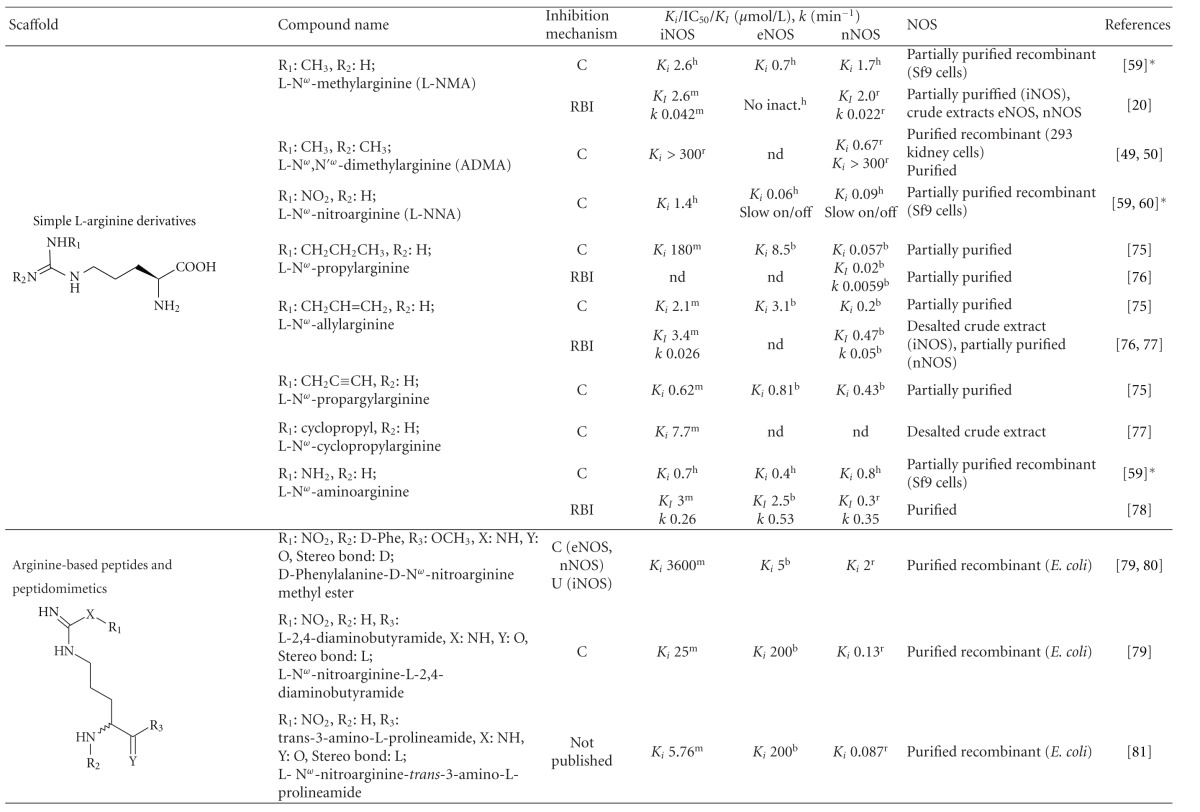 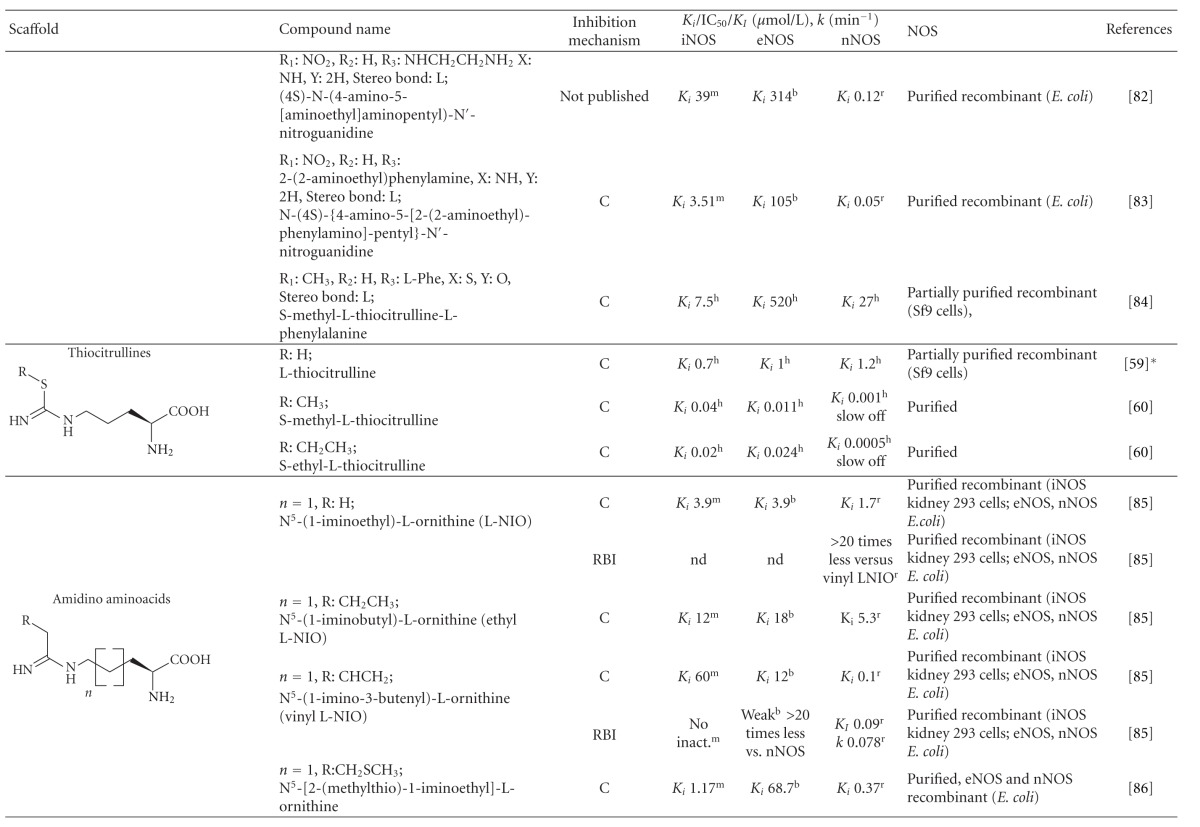 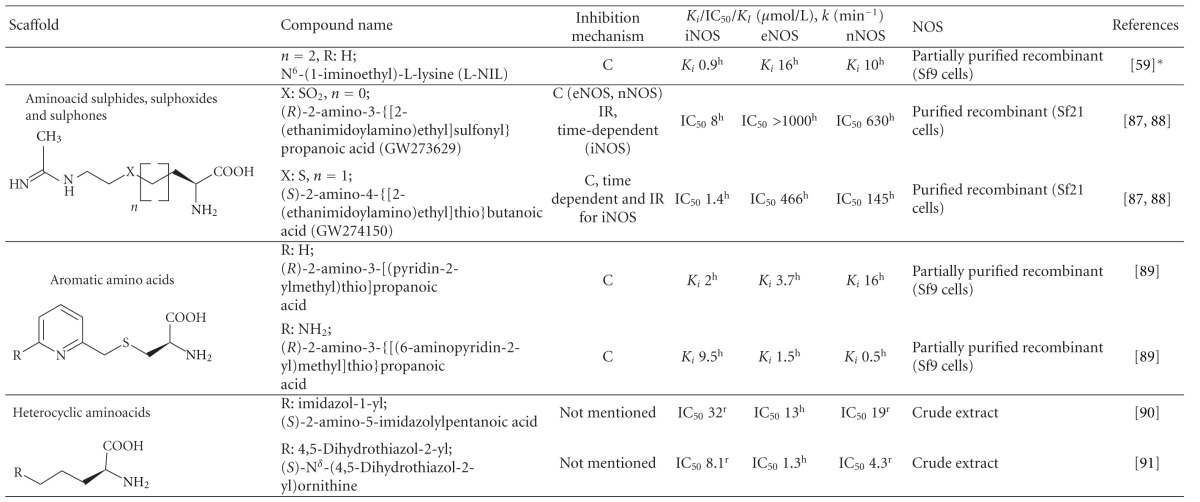

Inhibition mechanism: C: competitive, U: uncompetitive, IR: irreversible, RBI: reaction-based inhibitor; Inhibition constants: *K*_*i*_: inhibitor affinity constant (competitive inhibition), IC_50_: concentration at which 50% inhibition is achieved under specific conditions, *K*_*I*_: half maximal rate of enzyme inactivation, *k*: rate constant of enzyme inactivation, nd: data not available in the referred literature; enzyme origin: ^h^human, ^m^murine, ^r^rat, ^b ^bovine, *K_*i*_ values were obtained by recalculation using data in [59]. The extent of the inhibition can be estimated in the case of competitive inhibitors using the equation Inhibition  (%) = 100(*I*/(*I* + *K*_*i*_(1 + (Arg/*K*_*m*_))))   *I*: inhibitor concentration, Arg: arginine concentration, *K*_*m*_: Michaelis constant of the enzyme. *K*_*m*_ ranges (*μ*mol/L): iNOS^h^ 2.2–22, eNOS^h^ 0.9–4.4, nNOS^h^ 1.5–6.0, iNOS^m^ 2.3–14, eNOS^m^ 1.7–3.6, nNOS^m^ 1.3-1.4, eNOS^b^ 3.0–5.0, nNOS^b^ 2.0–3.3, iNOS^r^ 19–32, nNOS^r^ 1.5–14 [59, 79, 89, 92–97].

## Abbreviations

ADMA: L-N^*ω*^,N^*ω*^-dimethylarginine
BH_4_: (6R)-5,6,7,8-tetrahydrobiopterin
eNOS: Endothelial nitric oxide synthase
FAD: Flavin adenine dinucleotide
FMN: Flavin mononucleotide
GW273629: (2R)-2-amino-3-{[2-(ethanimidoylamino)ethyl]sulfonyl}propanoic acid
GW274150: (2S)-2-amino-4-{[2-(ethanimidoylamino)ethyl]thio}butanoic acid
IC_50_: Half maximal inhibitory concentration
iNOS: Inducible nitric oxide synthase
i.p.: Intraperitoneal
i.v.: Intravenous
*k*: Rate constant of enzyme inactivation
*K*_*i*_: Inhibitor affinity constant (competitive inhibition)
*K*_*I*_: Half maximal rate of enzyme inactivation
L-NAME: L-N^*ω*^-nitroarginine methyl ester
L-NIL: N^6^-(1-iminoethyl)-L-lysine
L-NIL-TA: N^6^-(1-iminoethyl)-L-lysine 5-tetrazole-amide
L-NIO: N^5^-(1-iminoethyl)-L-ornithine
L-NMA: L-N^*ω*^-methylarginine
L-NNA: L-N^*ω*^-nitroarginine
L-NPLA: L-N^*ω*^-propylarginine
L-SMTC: L-S-methylthiocitrulline
L-TC: L-thiocitrulline
NADPH: Nicotinamide adenine dinucleotide phosphate
nNOS: Neuronalnitric oxide synthase
NOS: Nitric oxide synthase
s.c.: Subcutaneous.
