# Supplementary material for: Arginine-Based Inhibitors of Nitric Oxide Synthase: Therapeutic Potential and Challenges
Source: Mediators Inflamm. 2012 Sep 4;2012:318087. doi: 10.1155/2012/318087 (PMC3441039; doi:10.1155/2012/318087)
Supplement: Supplementary file 1 — The supplementary material provides a list of recently designed clinical trials using arginine-based nitric oxide synthase inhibitors with unpublished results. [file 318087.f1.doc]

**Supplementary table 1:** List of recently designed clinical trials using L-NMA hydrochloride with unpublished results. Trial numbers at [www.clinicaltrials.gov](http://www.clinicaltrials.gov/) are given.

| **Use** | **Study name** | **Short descripion** | **Drug dosage** | **Number/Link** |
| --- | --- | --- | --- | --- |
| Global circulation | L-NMMA's Effect on Renal and Cardiovascular Variables in Healthy Subjects. A Randomized, Placebo-controlled Crossover Study | The aim is to investigate the effect of different levels of nitric oxide inhibition on blood pressure and arterial stiffness in healthy subjects | i.v.,  3 to 6 mg/kg priming bolus,  2 to 4 mg/(kg h) maintenantce for 1 hr | [NCT01070940](http://www.clinicaltrials.gov/ct2/show/NCT01070940?term=NCT01070940&rank=1) |
|  | Diesel Exhaust Inhalation, Systemic Nitric Oxide Inhibition and Cardiac Output | The goal of this study is to investigate the cardiovascular responses to systemic nitric oxide synthase inhibition following exposure to dilute diesel exhaust. | i.v.  3 mg/kg | [NCT01060930](http://www.clinicaltrials.gov/ct2/show/NCT01060930?term=NCT01060930&rank=1) |
| Hypertesnsion | Pathophysiology of Cardiometabolic Risk Factors in African Americans | The study is focused on determination the autonomic and nitric oxide contribution in the pathogenesis of hypertension and insulin resistance in obese African American women | Way not mentioned,  15 mg/(kg hr), | [NCT01122407](http://www.clinicaltrials.gov/ct2/show/NCT01122407?term=NCT01122407&rank=1) |
| Renal circulation | NO-Synthesis in Patients With Liver Cirrhosis: Effect of L-NMMA on Renal Hemodynamics, Sodium Excretion and Plasma Levels of Vasoactive Hormones | This is a study of the effects of nitric oxide inhibition with L-NMA in patients with liver cirrhosis and healthy controls | Not mentioned | [NCT00344916](http://www.clinicaltrials.gov/ct2/show/NCT00344916?term=NCT00344916&rank=1) |
|  | Effect of Elevated Plasma-Free-Fatty-Acids on Renal Hemodynamic Parameters | The aim of the present study is to characterise the hemodynamic effects of FFAs in the kidney. In addition we want to test the hypothesis that FFA-induced changes are mediated via endothelial derived nitric oxide (NO) | Not mentioned | [NCT00431665](http://www.clinicaltrials.gov/ct2/show/NCT00431665?term=NCT00431665&rank=1) |
|  | Effects of Systemic NO-Inhibition on Renal Hemodynamics in Patiens With Polycystic Kidney Disease and Chronic Glomerulonephritis | The study tests the hypothesis that systemic and renal nitric oxide availability is changed in polycystic kidney disease and chronic glomerulonephritis. | Not published | [NCT00345137](http://www.clinicaltrials.gov/ct2/show/NCT00345137?term=NCT00345137&rank=1) |
|  | Effects of Inhibition of NO-Synthesis on Renal Hemodynamics and Sodium Excretion in Patients With Essential Hypertension and Healthy Controls | The study tests the hypothesis that systemic and renal nitric oxide synthesis is changed in essential hypertension by investigating the effects of a non selective nitric oxide inhibitor on renal hemodynamics and sodium excretion in patients with essential hypertension. | Not published | [NCT00345150](http://www.clinicaltrials.gov/ct2/show/NCT00345150?term=NCT00345150&rank=1) |
|  | Effects of Acute L-NMMA Treatment on Renal Hemodynamics, Sodium and Water Excretion and Plasma Levels of Vasoactive Hormones in Patients With Congestive Heart Failure and Healthy Controls | The study tests the hypothesis that nitric oxide availability is changed in congestive heart failure with regard to the regulation of renal hemodynamics, renal sodium excretion and release of vasoactive hormones | Not published | [NCT00344734](http://www.clinicaltrials.gov/ct2/show/NCT00344734?term=NCT00344734&rank=1) |
| Ocular circulation | Role of Endothelin- and Nitric Oxide-System in the Regulation of Optic Nerve Head Blood Flow During Changes in Ocular Perfusion Pressure | The study determine the role of Endothelin- and Nitric Oxide-System in the Regulation of Optic Nerve Head Blood Flow During Changes in Ocular Perfusion Pressure | i.v.,  6 mg/kg bolus  0.06 mg/(kg min) for 12 min | [NCT00406731](http://www.clinicaltrials.gov/ct2/show/NCT00406731?term=NCT00406731&rank=1) |
|  | Role of Nitric Oxide in Optic Nerve Head Blood Flow Regulation During Experimental Increase of Intraocular Pressure in Healthy Humans | The present study is designed to test the hypothesis that NO plays a role in optic nerve head blood flow autoregulation during increased intraocular pressure. L-NMA serves as a tool to modulate NO level. | i.v.,  6 mg/kg bolus  0.06 mg/(kg min) for 15 min | [NCT00914394](http://www.clinicaltrials.gov/ct2/show/NCT00914394?term=NCT00914394&rank=1) |
|  | Role of Nitric Oxide in Optic Nerve Head Blood Flow Regulation During Isometric Exercise in Healthy Humans | The study is focuses on determination of the role of NO in the autoregulation of nerve head blood flow under increased systemic perfusion pressure. This condition is achieved by isometric execise (squatting). L-NMA is administered to modulate NO level. | i.v.,  6 mg/kg bolus  0.06 mg/(kg min) for 15 min | [NCT00806741](http://www.clinicaltrials.gov/ct2/show/NCT00806741?term=NCT00806741&rank=1) |
|  | Does eNOS Gene Polymorphism Play a Role in the Maintenance of Basal Vascular Tone in the Choroid or Optic Nerve Head | The goal of the study is to assess if the T -786C eNOS gene polymorphism ,which is thought to reduce eNOS activity, determines choroidal and optic nerve head blood flow. L-NMA is administered to modulate NOS activity | i.v.,  6 mg/kg bolus | [NCT00708357](http://www.clinicaltrials.gov/ct2/show/NCT00708357?term=NCT00708357&rank=1) |
|  | Mechanisms of Choroidal Blood Flow Changes During Dark/Light Transitions | Determination of the role of NO in Mechanisms of Choroidal Blood Flow Changes During Dark/Light Transitions | Not published | [NCT00431392](http://www.clinicaltrials.gov/ct2/show/NCT00431392?term=NCT00431392&rank=1) |

**Supplementary table 2:** List of recently designed clinical trials using L-NNA and L-NAME with unpublished results. Trial numbers at [www.clinicaltrials.gov](http://www.clinicaltrials.gov/) are given

| **Use** | **Study name** | **Short descripion** | **Drug dosage** | **Number/Link** |
| --- | --- | --- | --- | --- |
| Cardiac physiology | PET Detection of the Effects of Aging on the Human Heart. | The purpose of this study is to determine, with Positron Emission Tomography, the role of nitric oxide in the age-associated effect on fatty acid and glucose delivery on myocardial substrate metabolism. | i.v.,  4 mg/kg L-NAME | [NCT00603720](http://www.clinicaltrials.gov/ct2/show/NCT00603720?term=NCT00603720&rank=1) |
| Pulmonary physiology and Blood pressure | The Effect of Nitric Oxide on Pulmonary Resistances and Blood Pressure in Persons With Tetraplegia | The purpose is to determine if the levels of exhaled NO in individuals with chronic cervical spinal cord injury suffering from reduced baseline airway caliber and non-specific airway hyper-responsiveness is elevated to an extent like in able-bodied asthmatics. Further scientific conclusions about NO and its role in control of airway tone, pulmonary resistances and blood pressure will be drawn upon intravenous and inhaled administration of L-NAME. | Inhalation, nebulized 1 mg/kg L-NAME in saline;  i.v. 1-2 mg/kg L-NAME | [NCT00753948](http://www.clinicaltrials.gov/ct2/show/NCT00753948?term=NCT00753948&rank=1) |
| Blood pressure | Safety and Efficacy of L-NAME and Midodrine to Increase Mean Arterial Pressure in Persons With Tetraplegia | The aim is to determine how a spinal cord injury affects blood pressure and blood flow to the brain in a wheelchair seated patinent under medication with L-NAME and midodrine. | i.v.,  L-NAME 1 mg/kg | [NCT00835224](http://www.clinicaltrials.gov/ct2/show/NCT00835224?term=NCT00835224&rank=1) |
|  | Prevention of Low Blood Pressure in Persons With Tetraplegia | The aim of this investigation is to determine the blood pressure response to NOS inhibition with L-NAME in persons with tetraplegia with a special focus on such response in person in a seated posture. | i.v.,  L-NAME 1 mg/kg | [NCT00237770](http://www.clinicaltrials.gov/ct2/results?term=NCT00237770) |
| Local circulation | Role of Epoxy-eicosatrienoic Acids in Post-occlusive Hyperemia and Thermal Hyperemia | The objective of this study is to assess the involvement of epoxy-eicosatrienoic acids (EETs) in post-occlusive hyperemic and thermal hyperemia responses, and the interaction between nitric oxide (NO) and EETs. | microdialysis,  L-NAME 10 mmol/dm^3^ | [NCT01290198](http://www.clinicaltrials.gov/ct2/show/NCT01290198?term=NCT01290198&rank=1) |
| Solid tumor and local circulation | NG-Nitro-L-Arginine in Treating Patients With Advanced Solid Tumors | As NG-nitro-L-arginine may stop the growth of tumor cells by disrupting blood flow to the tumor. This trial is focused on studying the side effects and best dose of NG-nitro-L-arginine in treating patients with advanced solid tumors. | i.v., L-NNA  Dose not published | [NCT01324115](http://www.clinicaltrials.gov/ct2/show/NCT01324115?term=NCT01324115&rank=1) |

**Supplementary table 3:** List of recently designed clinical trials using GW274150 with unpublished results. Trial numbers at [www.clinicaltrials.gov](http://www.clinicaltrials.gov/) are given

| **Use** | **Study name** | **Short descripion** | **Drug dosage** | **Number/Link** |
| --- | --- | --- | --- | --- |
| Rheumatoid arthritis | An Open-Label, Single Dose Study to Investigate the Safety, Tolerability and Pharmacokinetics of GW274150 in Adult and Elderly Rheumatoid Arthritis Subjects. | This is a study of GW274150, an iNOS inhibitor to investigate safety, tolerability and pharmacokinetics in the rheumatoid arthritis population (greater than 50 years). | way not mentioned,  90 mg, single dose | [NCT00370435](http://www.clinicaltrials.gov/ct2/show/NCT00370435?term=NCT00370435&rank=1) |
|  | A Study To Investigate GW274150 Or Prednisolone In Rheumatoid Arthritis Taken Repeatedly For 28 Days | This is an exploratory study to examine the effect of iNOS inhibition in rheumatoid arthritis patients. The study involves 28 days repeat dosing with GW274150 (dose determined by the results from a previous study), prednisolone (7.5mg) or placebo. | way not mentioned,  60 mg/day for 28 days | [NCT00379990](http://www.clinicaltrials.gov/ct2/show/NCT00379990?term=NCT00379990&rank=1) |
